# General Hospital, Birmingham

**Published:** 1920-07-24

**Authors:** 


### General Hospital, Birmingham.

CHARGE FOR IN-PATIENTS.

THE Board of Management of the General Hospital, Birmingham, has recommended that the following steps be taken :—

The sale of securities to realise the sum of £50,000 to pay off the overdraft at the bank.

That from January 1, 1921, all in-patients be required to pay a maintenance charge of a guinea a week, and that children under twelve be charged half a guinea a week; but that the Board be empowered to remit all or part of the maintenance charge in respect of patients unable, in its opinion, to pay this.

The Board has also recommended that Law 12 be altered so that every annual subscriber of one guinea or more be entitled to receive three tickets for each guinea subscribed, and that each ticket bear the words "As regards the maintenance charge this ticket will be accepted in lieu of a cash payment of seven shillings Each ticket will be accepted as an out-patient ticket."

